# It Takes a Village: Multimodality Imaging of Cardiac Amyloidosis

**DOI:** 10.14797/mdcvj.1072

**Published:** 2022-03-14

**Authors:** Jean Michel Saad, Ahmed Ibrahim Ahmed, Dixitha Anugula, Yushui Han, Moath Said Alfawara, Mouaz H. Al-Mallah

**Affiliations:** 1 Houston Methodist DeBakey Heart & Vascular Center, Houston Methodist Hospital, Houston, Texas, US

**Keywords:** cardiac amyloidosis, cardiac magnetic resonance imaging, echocardiography, PET, positron emission tomography, cardiac scintigraphy, pyrophosphate

## Abstract

Cardiac amyloidosis (CA) is the buildup and infiltration of amyloid plaque in cardiac muscle. An underdiagnosed form of restrictive cardiomyopathy, CA can rapidly progress into heart failure. CA is evaluated using a multimodality approach that includes echocardiography, cardiac magnetic imaging, and nuclear imaging. Echocardiography remains an essential first-line modality that raises suspicion for CA and establishes functional baselines. Cardiac magnetic imaging provides additional incremental value via high-resolution imaging, robust functional assessment, and superior tissue characterization, all of which enable a more comprehensive investigation of CA. Cardiac scintigraphy has eliminated the need for invasive diagnostic approaches and helps differentiate CA subtypes. Positron emission tomography is the first modality introducing targeted amyloid binding tracers that allow for precise burden quantification, early detection, and disease monitoring. In this review, we highlight the role of several cardiac imaging techniques in the evaluation of CA.

## Introduction

**Figure 1:**
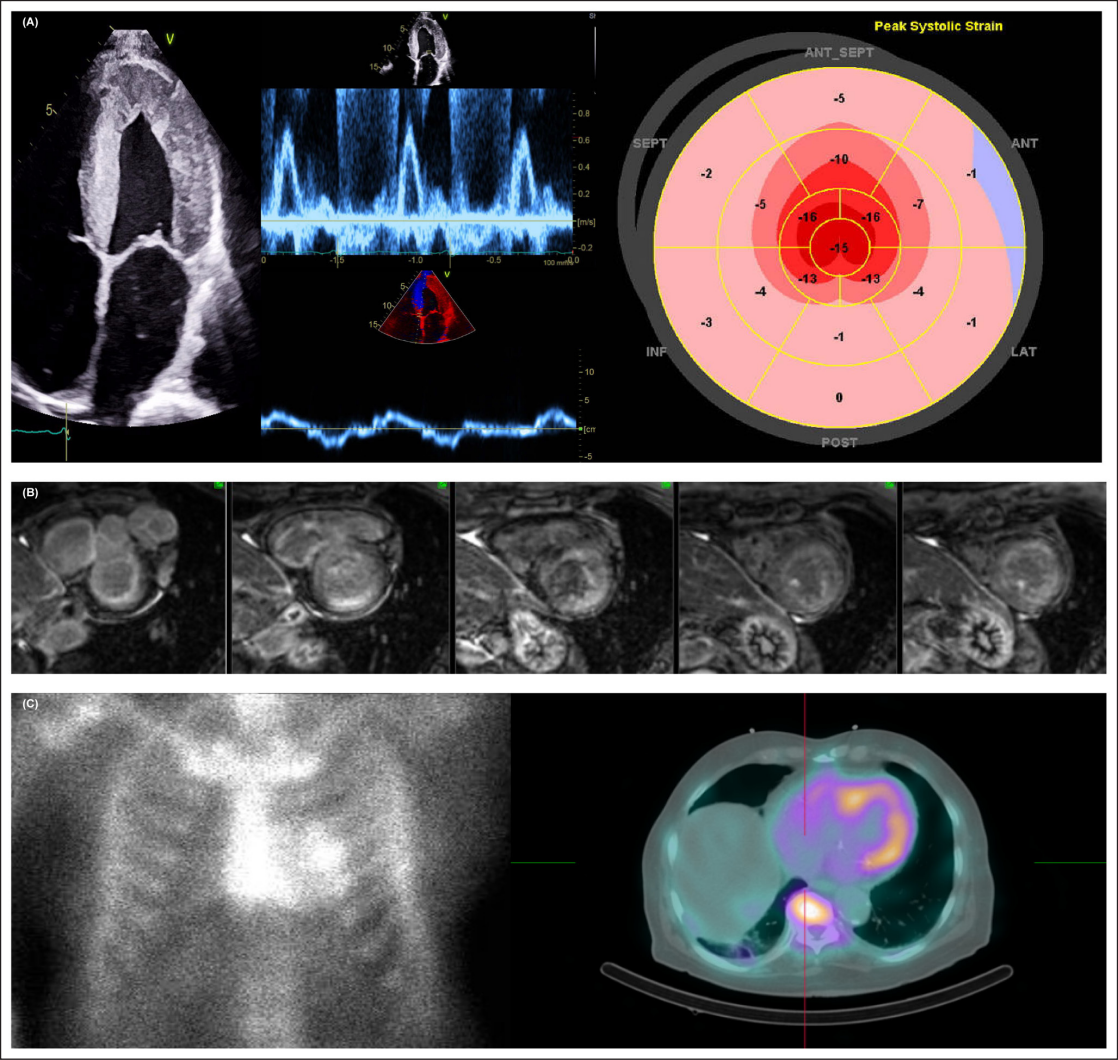
Multimodality imaging approach for cardiac amyloidosis (CA). **(A)** Increased left ventricular (LV) wall and interatrial thickness with sparkling texture are typically found in patients with CA (left). Evidence of restrictive LV filling, rapid E-wave deceleration time, high E/A ratio on mitral inflow Doppler, and low myocardial relaxation velocities on tissue Doppler imaging (middle). Corresponding “bullseye” map of the longitudinal strain pattern of the LV with a “cherry on top” sign (right). **(B)** Cardiac magnetic resonance imaging shows characteristic imaging of CA with diffuse and subendocardial late gadolinium enhancement. **(C)** ^99m^Tc-PYP anterior planar (left) imaging and single-photon emission computed tomography/computer tomography (right) showing a Perugini Grade of 2 and 3, respectively.

Cardiac amyloidosis (CA) is a clinical condition in which one of more than 30 different precursor proteins with unstable tertiary structure misfolds and aggregates as insoluble amyloid fibrils and deposits in the extracellular space of the heart.^1^ CA leads to restrictive cardiomyopathy that predominantly presents as right ventricular (RV) failure. The workup and assessment of cardiac amyloidosis have employed a wide range of multimodality imaging techniques, each offering unique insights (***Figure 1***). This review discusses the role of several cardiac imaging techniques—including echocardiography, cardiac magnetic imaging, cardiac scintigraphy, and positron emission tomography (PET)—in the workup of CA.

## Echocardiography

From M-mode findings almost 40 years ago, echocardiography has become a standard in any diagnostic evaluation of cardiac amyloidosis.^2^ Echocardiography’s widespread availability, accessibility, and low cost make it the first noninvasive imaging modality used when investigating the suspicion of CA.^3^ Although not sensitive or specific enough to confirm a diagnosis, echocardiography provides critical information regarding disease status, cardiac function, and follow-up in the setting of good image quality and a high index of suspicion.^4,5,6,7^

Cardiac amyloidosis develops following increased deposition and accumulation of amyloid fibrils specifically in the ventricular walls. These depositions translate into macro changes that form the basis of echocardiographic morphological assessment. These changes most prominently manifest as a thickened intraventricular septal wall > 12 mm that, in the absence of other causes of left ventricular (LV) hypertrophy, would indicate cardiac involvement in amyloid light-chain (AL) systemic amyloidosis.^8^ However, the ability to accurately differentiate cardiac amyloidosis from other cardiomyopathies remains extremely difficult and highlights a recurrent limitation of echocardiography. Echocardiography can detect classical changes including thickening of the ventricular walls, interatrial septum, and valves, resulting in a smaller LV cavity, biatrial enlargement, and restrictive LV filling pattern.^9,10,11,12^ Another reported finding includes a granular sparkling appearance of the myocardial wall, although this also was observed in other disease processes, namely end-stage renal disease.^13^ More importantly, these changes are typically seen in more advanced stages of CA, hence limiting the utility of echocardiography in identifying early CA.

Echocardiography excels at providing exclusive insight into diastolic function and LV filling pressures. A high A-wave, low E-wave, and subsequently a reduced E/A ratio are indicative of possible early-stage CA compared with low A-wave and normal E-wave leading to a high E/A ratio in late CA.^14^ Advanced restrictive patterns also include a rapid decelerating diastolic MV inflow time and small S-wave pulmonary venous spectral Doppler patterns. Furthermore, reduced mitral and tricuspid annular velocities and a high E/e’ ratio are also indicative of increased filling pressures.^15^

Cardiac amyloidosis has exhibited a progressive pattern of diastolic dysfunction characterized by impaired relaxation. Following a stage of preserved ejection fraction,^16,17^ these patterns ultimately lead to a deterioration of LV function.^18^ Similar patterns are also noted using other functional assessment parameters such as stroke volume index and myocardial contraction fraction (MCF), which have both proven to be better diagnostic markers than ejection fraction.

Moreover, stroke volume index is easily obtained and prognostically useful in patients with AL-CA, making it an essential parameter in the CA workup.^17^ MCF is a ratio of stroke volume to myocardial volume, thus creating an index that assesses volumetric shortening of the myocardium independent of chamber size and geometry.^19,20,21^

Although most conventional echocardiographic parameters report low diagnostic accuracy due to low sensitivity, other parameters have shown remarkable specificities. Some examples include E/e’ ratio (> 9.6 specificity is 100%, and sensitivity 50%), left atrial volume index (≥ 47 mL/m^2^ specificity is 93%, and sensitivity 44%), and MCF (≤ 0.234 specificity is 96%, and sensitivity 56%).^22^

The recent implementation of Doppler imaging and speckle-tracking echocardiography has introduced longitudinal strain measurement as a useful assessment of quantitative systolic function.^11,23,24^ Using 2-dimensional (2D) speckle tracking, abnormal myocardial deformation has been detected in as many as 93% to 100% of patients with CA.^11^

Moreover, both transthyretin amyloidosis (ATTR) and AL cardiac amyloidosis are known to lead to reduced global longitudinal strain prior to any noticeable changes in left ventricular ejection fraction. However, this is more prominent in the detection of ATTR cardiomyopathy (ATTR-CA), with a global longitudinal strain of -15.1 or greater reporting an area under the curve (AUC) of 0.85 (87% sensitivity and 72% specificity).^22^ Furthermore, a regional gradient pattern showing impairment of the mid and basal segments with relative sparing of the apex— known as the “bullseye”—is consistent with cardiac amyloidosis.^25,26^ Although the underlying pathophysiology remains unclear, this pattern has allowed clinicians to differentiate between cardiac amyloidosis and other causes of LV hypertrophy, such as hypertensive cardiomyopathy or aortic stenosis, which typically present with reduced LV longitudinal strain in the areas of maximum hypertrophy.^26,27^ An apical/average longitudinal strain ratio > 1.0 in the mid and basal segments was able to distinguish CA from LV hypertrophy (93% sensitivity, 82% specificity).^26^ Reduced global longitudinal strain carries significant prognostic implications in both AL amyloidosis and ATTR.^11,25,28^

## Cardiac Magnetic Resonance

Improved technology and heightened provider awareness have led to the increased use of cardiac magnetic imaging (CMR) in the workup of cardiac amyloidosis.^29^ CMR provides additional incremental value in the form of high-resolution imaging, robust functional assessment, and superior tissue characterization, enabling a more comprehensive investigation of CA. The use of CMR is therefore crucial to the continued assessment of CA, especially in cases of poor acoustic windows or uncertain diagnosis following echocardiography. In addition to anatomical and functional assessment, CMR offers an array of other parameters in the workup of CA, including T1/T2-weighted imaging, T1/T2 mapping, late gadolinium enhancement (LGE), and extracellular volume (ECV).^3^

As previously noted on echocardiography, CA was known to cause LV hypertrophy across AL amyloidosis and ATTR-CA. However, owing to its high image resolution and 3D image acquisition, CMR noted that concentric symmetrical LV hypertrophy is more typical of AL amyloidosis, occurring in approximately 79% of patients. Conversely, 68% of patients with ATTR-CA have asymmetrical hypertrophy compared with 18% of those with AL amyloidosis,^30^ while no differences were noted among the subtypes of ATTR-CA: wild-type ATTR and hereditary ATTR. In addition, amyloid deposition in the RV wall results in a typical RV hypertrophy morphology.

**Figure 2:**
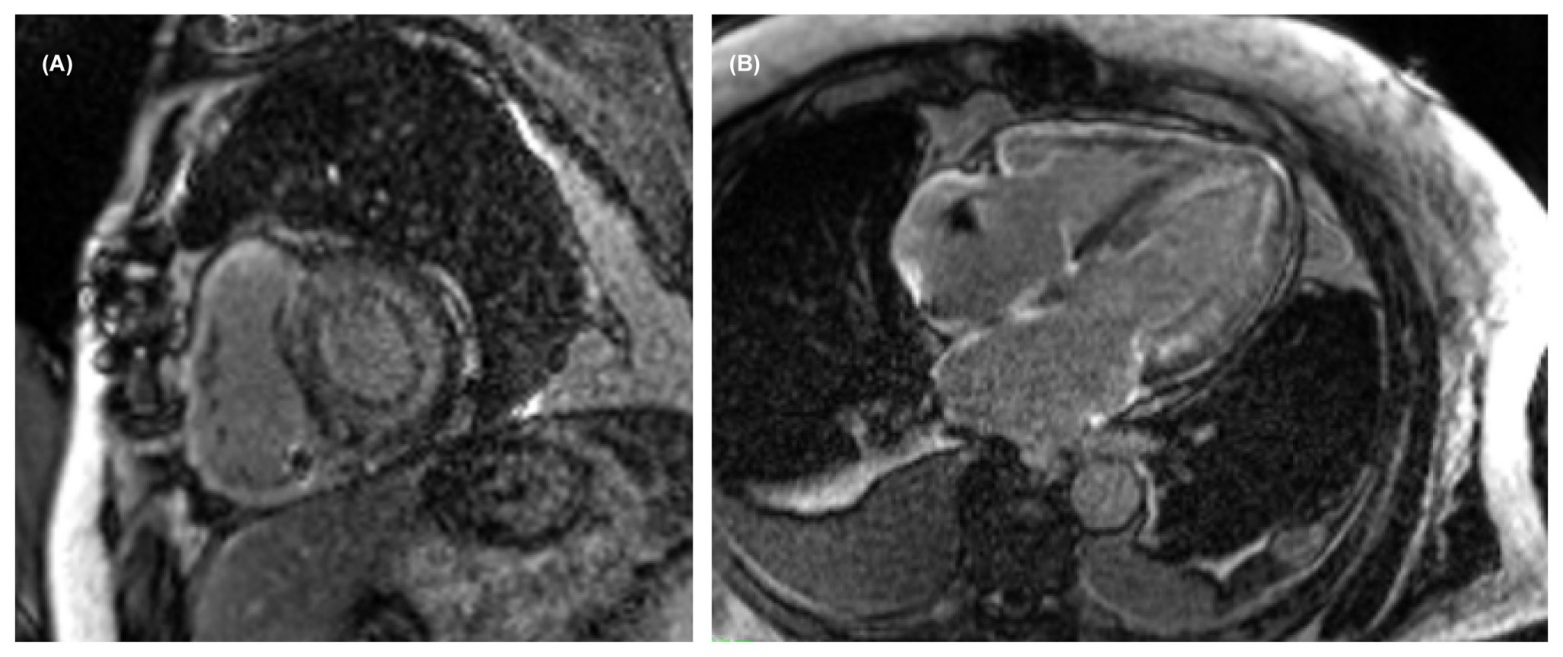
**(A)** Short-axis view and **(B)** long-axis view shows diffuse late gadolinium enhancement uptake (Seimens 1.5 Tesla).

Another unique advantage offered by CMR is LGE, a highly accurate finding for the detection of CA (***Figure 2***).^31,32^ First described by Maceira et al., global subendocardial LGE became a pathognomonic finding highly suggestive of CA.^31^ Furthermore, LGE in the left atrium is specific for differentiating cardiac amyloidosis from other hypertrophic cardiomyopathies.^33^

Initial implementation of LGE proved technically challenging due to difficulty with myocardial nulling. However, the use of a widely available phase-sensitive inversion recovery sequence (PSIR) helped resolve previous issues with optimal nulling settings and served to increase the reliability and application of LGE CMR.^31^ LGE was also used to track progression in both AL and ATTR amyloidosis based on progression from typical subendocardial to transmural enhancement.^34,35^ Although other LGE patterns were described, RV, subendocardial, and transmural remained the most commonly reported.^30^ Studies comparing LGE patterns between ATTR and AL amyloidosis noted that while both forms exhibited similar LGE patterns, a higher prevalence of subendocardial LGE was reported in AL amyloidosis compared with transmural and RV LGE in ATTR amyloidosis.^30,35,36,37^

Although LGE is a qualitative indicator of CA, it lacks the ability to quantitatively measure amyloid infiltration due to the large heterogeneity in pattern and signal intensities. Furthermore, LGE utilization is limited in patients with renal impairment, a subsequent manifestation in amyloidosis patients.^38^

**Figure 3:**
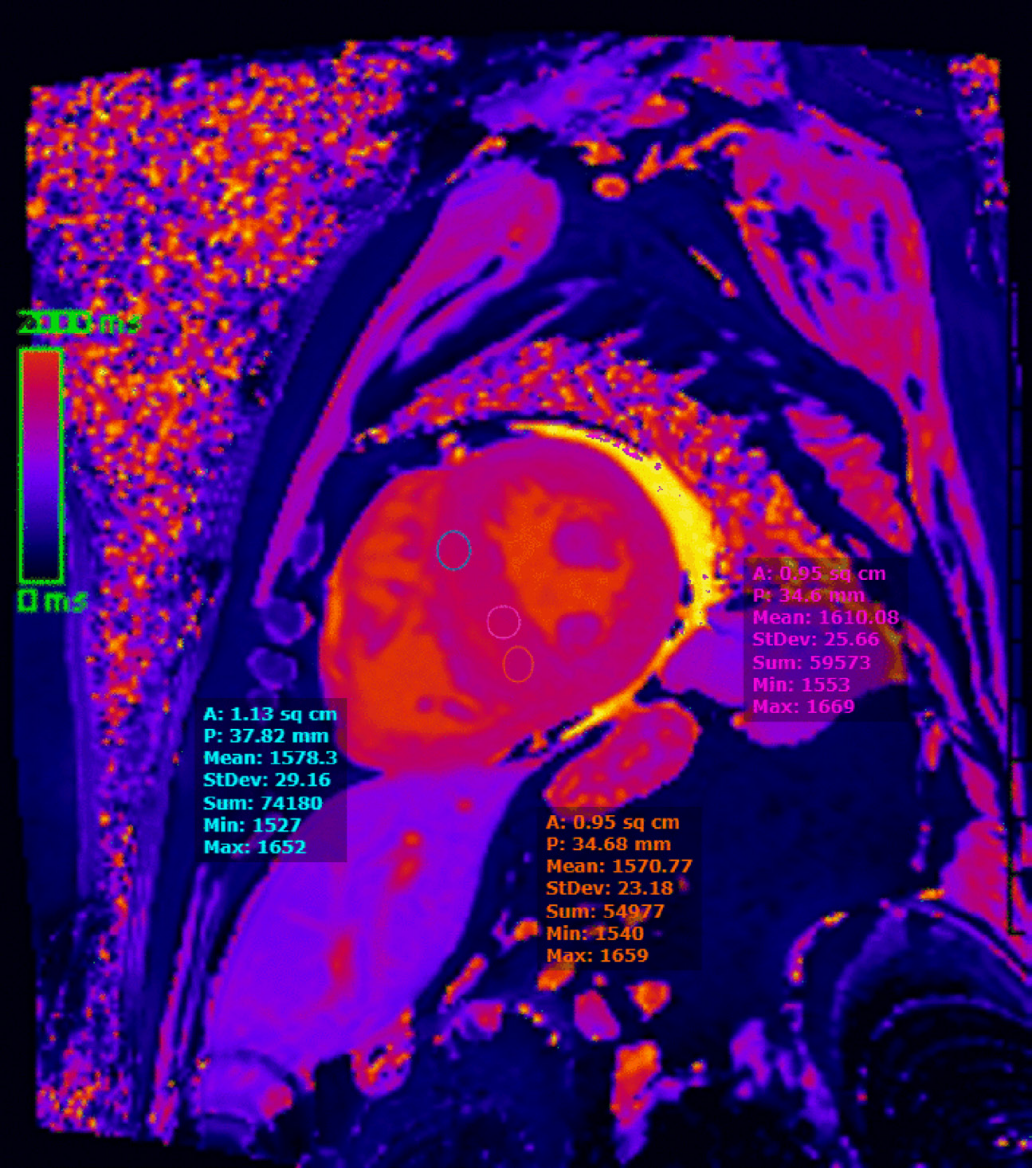
T1 map (precontrast) shows the left ventricle with an elevation of native T1 values (Seimens 1.5 Tesla).

In contrast, T1 and ECV provide benchmarks to quantify and track disease burden, allowing for disease stratification and evaluation of response to therapy.^39^ T1- and T2-weighted imaging sequences are intrinsic magnetic parameters based on the abundance and magnetization of hydrogen nuclei in tissues. Native (noncontrast) T1 mapping is a composite signal from both extra and intracellular spaces (***Figure 3***).^40,41^ The addition of contrast (LGE) accentuates these properties and allows for the isolation and measurement of ECV, categorizing T1 mapping as precontrast (native T1) and postcontrast (ECV). Native myocardial T1 is a measurement of myocardial T1 relaxation times using noncontrast CMR, noting increased T1 values in both AL and ATTR amyloidosis.^42^ Similar diagnostic accuracy for detecting ATTR amyloidosis was reported by Fontana et al., albeit with a lower T1 elevation compared with AL amyloidosis.^43^ Studies have also suggested the use of native T1 as a marker of early cardiac involvement.^42,43^

Because amyloid deposition results in the gradual expansion of the extracellular matrix, the quantification of ECV can potentially provide insight into disease burden and degree of amyloid infiltration. Several studies reported an increase in ECV in both AL and ATTR amyloidosis.^39,40,41^ More importantly, studies have shown how an increase in ECV can serve as an early marker of disease since it is identified before LGE and abnormal findings in other imaging modalities.^44^

Higher native myocardial T1 mapping was able to identify and stratify worse prognosis in AL-CA but not in ATTR-CA.^45,46^ On the other hand, ECV has shown prognostic association in both AL and ATTR-CA. Ultimately, incremental to traditional risk factors, both LGE and ECV can serve as independent predictors of disease prognosis.^30,45,47^

CMR myocardial T2 mapping provides another dimension of tissue characterization by visualizing and quantifying myocardial edema. Prior studies have revealed elevated T2/myocardial edema in patients with acute myocardial infarction, myocarditis, and heart failure.^48,49^ Although T2 ratios in CA reported conflicting results, T2 mapping has shown consistent elevation in both ATTR and AL-CA. Furthermore, T2 was also shown to be an independent predictor of mortality even after adjusting for ECV and NT-proBNP.^50^

## Technetium-Labeled Cardiac Scintigraphy

Cardiac scintigraphy provides incremental value in the workup of CA because it can differentiate between AL and ATTR amyloidosis.^3,51^ More importantly, ^99m^Tc-labeled radiotracer cardiac scintigraphy prevents the need for invasive diagnostic procedures in patients screened for monoclonal gammopathies, effectively rewriting previous diagnostic approaches to CA, especially ATTR-CA.^3,52^ Initial interest in ^99m^Tc-labeled bone scans as an imaging tool for amyloidosis began during the 1970s and 1980s after studies reported diffuse myocardial uptake, particularly in the right and left ventricles of patients with CA.^53,54,55,56,57^ However, the potential of cardiac scintigraphy was not fully realized until the last two decades, following reports of improved diagnostic accuracy for ATTR-CA.^58,59,60^

**Figure 4:**
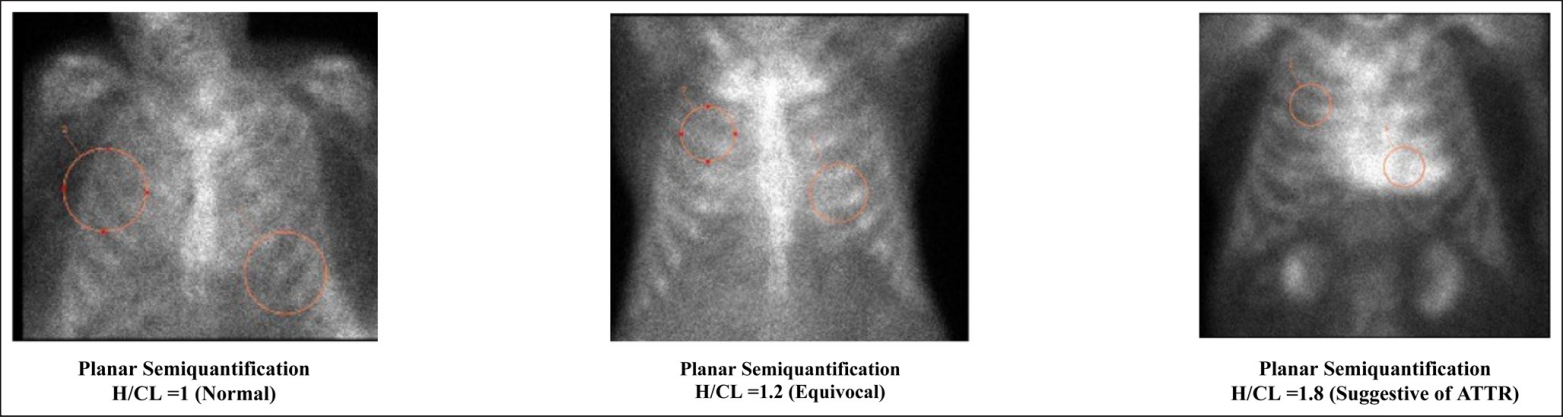
^99m^Tc-PYP cardiac scintigraphy shows anterior planar imaging using heart-to-contralateral ratio semiquantitative scoring. Reproduced with permission from Springer Nature. doi: *10.1007/s10741-021-10174-x*. H/CL: heart to contralateral lung; ATTR: transthyretin amyloidosis

**Figure 5:**
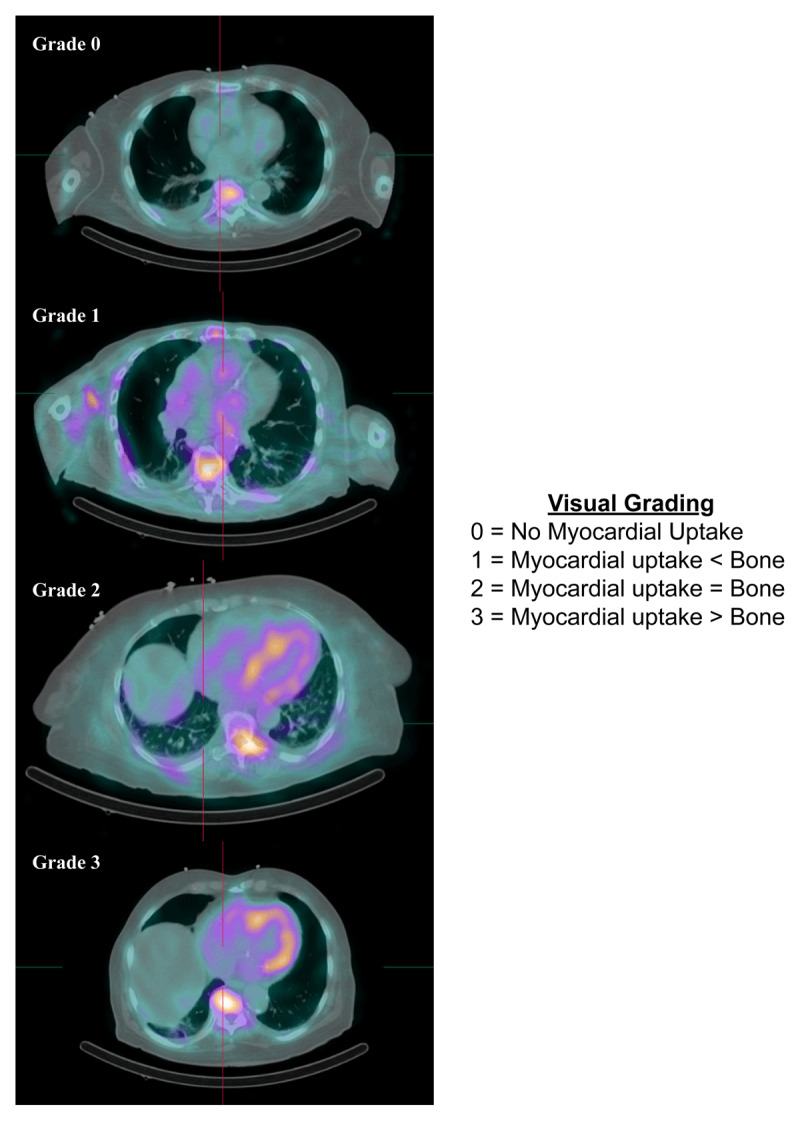
^99m^Tc-PYP cardiac scintigraphy using single photo emission computer tomography/computed tomography visual scoring. Reproduced with permission from Springer Nature. doi: *10.1007/s10741-021-10174-x*

Two grading systems are used for radiotracer uptake in single-photon emission computed tomography (SPECT) imaging, offering a semiquantitative and quantitative approach to CA workup. Heart to contralateral lung (H/CL) was used to quantify uptake by comparing radiotracer retention, where an H/CL ratio > 1.5 was suggestive of ATTR-CA (***Figure 4***). Perugini et al. established a semiquantitative visual system to score planar images obtained at 3 hours post injection. This system was based on the uptake comparison between bone (rib) and the heart. The scoring system proposed was as follows: 0 = absent cardiac uptake, 1 = uptake less than bone, 2 = uptake equal to bone, and 3 = uptake greater than bone (***Figure 5***).^58^

Applying this visual scoring system revealed that any grade (1,2, or 3) conferred a sensitivity of > 99% but low specificity of 68% when compared with EMBs to identify ATTR. However, following the screening of patients for monoclonal gammopathy, a grade ≥ 2 established a sensitivity and specificity of 100% along with a 100% positive predictive value reproduced along all 3 radiotracers.^52^ This obviated the need for more invasive confirmatory testing in patients with unexplained heart failure, suggestive echocardiography/CMR findings, and ruled out monoclonal gammopathy. Furthermore, a visual grade ≥ 2 also allowed the accurate differentiation of ATTR-CA from AL-CA (or unaffected controls). This is particularly relevant in the era of the new targeted amyloidosis treatments unique for both ATTR-CA and AL-CA.

The commonly investigated technetium-labeled radiotracers included ^99m^Tc-pyrophosphate (^99m^Tc-PYP), ^99m^Tc-3,3-diphosphono-1,2-propanodicarboxylic acid (^99m^Tc-DPD), ^99m^Tc-hydroxymethylene diphosphonate (^99m^Tc-HMDP), and ^99m^Tc methylene diphosphonate (^99m^Tc-MDP). ^99m^Tc-PYP is commonly used in the United States (US), while ^99m^Tc-DPD and ^99m^Tc-HMDP are used in Europe. On the other hand, ^99m^Tc-MDP, a widely available radiotracer in the US, is not routinely used due to its low sensitivity for detecting ATTR.^61^

It is also important to note that some specific hereditary ATTR mutations have shown varying uptakes and sensitivities in cardiac scintigraphy. In particular, V30M,^62,63^ Y114C,^63^ Thr59Lys,^59^ and Phe64Leu^64^ are reported as having low sensitivity with cardiac scintigraphy or no uptake with different Tc-labeled tracers (DPD and PYP) (***Table 1***). This discrepancy is due to specific TTR fibril compositions that include type A or type B (fragmented and full length versus exclusively full length, respectively),^65,66^ with full-length fibrils exhibiting little to no radiotracer uptake. To prevent missed diagnoses, a high degree of suspicion and a multimodality imaging approach are needed to balance out the inconsistencies presented by these mutations.

**Table 1:** Reported non-transthyretin amyloidosis causes of false positive and false negative Tc-labeled cardiac scintigraphy. Reproduced with permission from Springer Nature, doi: *10.1007/s10741-021-10174-x*


FALSE POSITIVE	FALSE NEGATIVE

Amyloid light-chain amyloidosis	Early disease

Hydroxychloroquine toxicity	Delayed imaging

Rib fracture	Full length (type B, Phe64Leu, Ser77Tyr, and Thr59Lys) transthyretin fibrils

Pleural effusion	Small radiotracer injection dose

Valvular calcification	Short acquisition time

Blood pool	Pericardial effusion

Breast implants	Breast implants

Myocardial infarction (acute or subacute)	

Rare forms of cardiac amyloidosis	

APO-A1 mutation	


In addition to its diagnostic utility in the workup of CA, cardiac scintigraphy has also proven useful in some avenues of CA prognostication. Particularly, an H/CL ratio > 1.6 was shown to predict worse survival among patients with ATTR-CA.^67^ Radiotracer retention grading and indices along different bone radiotracers have a reported association with cardiac biomarkers such as NT-proBNP, cardiac troponin T, and extracellular and other imaging parameters such as volume fraction, ejection fraction, LV wall thickness, and mass. Subsequently, radiotracer retention also has been associated with increased adverse cardiac outcomes such as advanced heart failure and mortality.^67,68,69,70,71^

## Positron Emission Tomography

Despite the attributes of cardiac scintigraphy, there continue to be gaps in the management of CA, such as the inability to clearly detect early disease as well as monitor disease progression and/or response to therapy. To counter these limitations, positron emission tomography (PET) has emerged as a new modality in the diagnostic evaluation of CA. PET offers several promising tracers such as Pittsburg Compound B, florbetapir, and florbetapan.^72^ While these tracers were originally created to visualize and quantify beta-amyloid plaques in patients with Alzheimer’s disease, they have since proven effective in diagnosing CA. Antoni et al. showed how there was a significant increase in ^11^C-PiB (Pittsburgh Compound B) retention index (RI) in patients with ATTR-CA and AL-CA compared to controls.^73^ A subsequent study showed significant differences in maximal and mean myocardium-to-blood cavity standard uptake value (SUV) ratios between AL-CA patients with prior chemotherapy versus chemotherapy naïve patients. This established the role of ^11^C-PiB as a surrogate indicator of active myocardial light chain deposition.^74^ Rosengren et al. showed how CA without cardiac wall hypertrophy had increased ^11^C-PiB uptake, indicating a possible role in early diagnosis before the development of overt morphological changes.^75^

^18^F-florbetapir and ^18^F-florbetapan, PET tracers of the Stilbene class, could serve as molecular imaging biomarkers for CA as they bind directly to amyloid fibrils.^76^ Earlier studies confirmed significant uptake of ^18^F-florbetapir in CA patients with a predilection for AL-CA compared to ATTR-CA.^77^ Similarly, 18F-florbetapan has the potential to differentiate between non-CA, AL-CA, and ATTR-CA. Furthermore, ^18^F-florbetapan retention in CA was an independent determinant of functional and morphological parameters cardiac dysfunction on echocardiogram (for global LV longitudinal strain) and CMR imaging (for ventricular wall thickness).^78,79^

However, some new limitations arise with the use of these PET tracers—mainly, the shorter half-life of the ^11^C-PiB radiotracer versus ^18^F-labeled PET tracers. This necessitates the installation of an on-site cyclotron for continuous production, which may limit its accessibility and adoption.^75^ Moreover, these radiotracers are not yet approved for clinical use and are only available in limited specialized centers.

PET imaging has brought forth new targeted amyloid-binding radiotracers that facilitate detection of all amyloid deposits regardless of the particular subtype of CA. More importantly, early studies have suggested that, using ^11^C-PiB and ^18^F-florbetapir, PET imaging may detect CA well before macroscopic changes such as wall thickening or later changes in cardiac biomarkers.^75,80^ As such, PET introduces new avenues in the early diagnosis, monitoring, and assessment of CA burden.

## Conclusion

Multimodality imaging tools provide complementary roles in the diagnosis of patients with suspected CA. Echocardiography is the first-line test to identify nonspecific signs such as ventricular wall thickening and restrictive filling patterns. CMR enables high-resolution structural assessment as well as qualitative and quantitative measurement of amyloid infiltration. Pyrophosphate cardiac scintigraphy’s strength is its ability to differentiate AL from ATTR amyloidosis. PET has an emerging role in early detection, disease monitoring, and response to therapy.

## Key Points

- Cardiac amyloidosis (CA) remains an underdiagnosed cause of rapidly progressive heart failure. The evaluation of CA entails a multimodality imaging approach encompassing echocardiography, cardiac magnetic resonance (CMR), and nuclear imaging.
- The combination of echocardiography and CMR provides valuable anatomical and functional cardiac assessment in an effort to increase suspicion and maximize diagnostic accuracy.
- ^99m^Tc-labeled radiotracer cardiac scintigraphy excels at differentiating and confirming the diagnosis of transthyretin CA following adequate screening and ruling out light-chain amyloidosis, obviating the need for more invasive biopsy approaches.
- The application of quantitative advanced cardiac imaging such as CMR and positron emission tomography have opened up new avenues in the early detection and prognostication of CA and tracking of therapeutic response, which is especially useful given the promising advancements in the treatment of CA.
